# Priority setting in early childhood development: an analytical framework for economic evaluation of interventions

**DOI:** 10.1136/bmjgh-2022-008926

**Published:** 2022-06-20

**Authors:** Stéphane Verguet, Sarah Bolongaita, Anthony Morgan, Nandita Perumal, Christopher R Sudfeld, Aisha K Yousafzai, Günther Fink

**Affiliations:** 1 Department of Global Health and Population, Harvard T.H. Chan School of Public Health, Boston, MA, USA; 2 University of Basel, Basel, Switzerland; 3 Swiss Tropical and Public Health Institute, Allschwil, Switzerland

**Keywords:** child health, public health, health economics

## Abstract

**Background:**

Early childhood development (ECD) sets the foundation for healthy and successful lives with important ramifications for education, labour market outcomes and other domains of well-being. Even though a large number of interventions that promote ECD have been implemented and evaluated globally, there is currently no standardised framework that allows a comparison of the relative cost-effectiveness of these interventions.

**Methods:**

We first reviewed the existing literature to document the main approaches that have been used to assess the relative effectiveness of interventions that promote ECD, including early parenting and at-home psychosocial stimulation interventions. We then present an economic evaluation framework that builds on these reviewed approaches and focuses on the immediate impact of interventions on motor, cognitive, language and socioemotional skills. Last, we apply our framework to compute the relative cost-effectiveness of interventions for which recent effectiveness and costing data were published. For this last part, we relied on a recently published review to obtain effect sizes documented in a consistent manner across interventions.

**Findings:**

Our framework enables direct value-for-money comparison of interventions across settings. Cost-effectiveness estimates, expressed in $ per units of improvement in ECD outcomes, vary greatly across interventions. Given that estimated costs vary by orders of magnitude across interventions while impacts are relatively similar, cost-effectiveness rankings are dominated by implementation costs and the interventions with higher value for money are generally those with a lower implementation cost (eg, psychosocial interventions involving limited staff).

**Conclusions:**

With increasing attention and investment into ECD programmes, consistent assessments of the relative cost-effectiveness of available interventions are urgently needed. This paper presents a unified analytical framework to address this need and highlights the rather remarkable range in both costs and cost-effectiveness across currently available intervention strategies.

**WHAT IS ALREADY KNOWN ON THIS TOPIC**

- No standardised framework exists that allows a comparison of the relative cost-effectiveness of early childhood development (ECD) interventions.

**WHAT THIS STUDY ADDS**

- We present an economic evaluation framework that focuses on the impact of ECD interventions on motor, cognitive, language and socioemotional skills, which we apply to compute the relative cost-effectiveness of ECD interventions.

**HOW THIS STUDY MIGHT AFFECT RESEARCH, PRACTICE AND/OR POLICY**

- Our framework enables direct value-for-money comparison of ECD interventions across settings.

## Introduction

Providing children under the age of 5 years with a supportive, nurturing and stimulating environment has proven to be of central importance for supporting positive human capital outcomes in later life.^1^ As summarised by Aboud and Yousafzai,^2 3^ ‘children who do not acquire a good vocabulary in the early years will have difficulty learning how to read; children who do not acquire simple problem-solving strategies in the first 24 months will have difficulty understanding math concepts; and children who do not develop secure emotional attachments to adults will have difficulty coping with stresses and challenges throughout life’.^2 3^

Mounting research points to early childhood during the first 5 years of life as a sensitive period for positively affecting long-term trajectories through interventions providing early learning opportunities, safe and supportive home environments and responsive and nurturing care.^4^ As a result, early childhood development (ECD) was included in the 2015 Sustainable Development Goals (SDGs). SDG Target 4.2 demands that ‘by 2030, all girls and boys have access to quality care for ECD and preprimary education so that they are ready for primary education’.^5^ Given that over 250 million children under 5 years of age are currently estimated to be at risk of not reaching their developmental potential in low-income and middle-income countries (LMICs),^6–8^ this is an ambitous task.

Even though attention for programmes that promote ECD has increased over the past few years,^9^ the resources available currently are without doubt insufficient to provide comprehensive programmes to all children globally. This naturally raises the question about which programmes should be prioritised. Remarkably, there is currently no framework that can provide guidance on this. Most of the existing literature on interventions that promote ECD has focused on the long-term benefits (or ‘returns to’), such as higher earnings, improved health, lower crime rates, increased productivity and other benefits for society as a whole^6 10^; and highlighted the generally high benefit-cost ratios of the programmes analysed. Even though these estimated benefit-cost ratios have been of critical importance for garnering political and donor interest in ECD programmes, currently available estimates are mostly based on a handful of relatively older intervention efficacy studies with relatively small sample sizes and long-term follow-up data and thus do not allow a general comparison or ranking of interventions currently available at the global scale.^6 11 12^ As pointed by Batura and colleagues in their review of the literature,^13^ few publications exist on the cost-effectiveness of interventions that promote ECD, especially from LMICs.

The development of standardised costing tools has been an important first step towards making interventions comparable across settings^14^ and now increasingly allows the calculation of programme costs at scale on a national level^15^ as well as towards identifying funding gaps for a wide range of early childhood intervention scenarios.^16 17^ What remains lacking is an analytical approach that allows researchers and policymakers to link cost to impact data and to create the value-for-money estimates needed for national and global priority setting.^18^ In this paper, we present a first framework for creating such estimates and use it to generate a first ranking of interventions with proven positive impact on ECD. While this framework and ranking are subject to several important limitations, which we discuss in further detail below, we view this work as a first step towards developing a unified priority setting framework for decision-making and eventual rollout of programmes that promote ECD globally.

## Methods

We first reviewed the existing literature to document the main approaches that have been used to assess the relative effectiveness of interventions that promote ECD. Second, interpreting this first scoping review, we present a basic economic evaluation framework that builds on these reviewed approaches, but focuses more directly on the immediate impact of interventions on early childhood outcomes. Third, we applied the framework to compute the relative effectiveness and cost of a range of interventions for which recent effectiveness and costing data were published,^2 3 14 19^ drawing from the review by Aboud and Yousafzai^19^ (see details below).

In order to identify effective interventions (that is interventions that directly or indirectly improve ECD), we first reviewed the literature (in a non-systematic manner) on ‘ECD-specific’ interventions, that is, programmes that were designed to directly impact on ECD outcomes. We then gathered impact (effectiveness) and cost data. Publications were collected through expert consultation or by searching academic websites (eg, PubMed, Google Scholar database): the PubMed database was searched with keywords like ‘cost-effectiveness+early childhood development’. However, due to the paucity of data on cost and effectiveness and the lack of comparability across studies and settings emerging from this initial scoping review, we then resorted to drawing from the review by Aboud and Yousafzai^19^ from which we extracted all relevant studies (ie, early parenting and at-home psychosocial stimulation interventions) for which effect sizes were documented in a consistent manner. The studies retained included parenting interventions that comprised at-home stimulation and responsive caregiving components designed to improve developmental outcomes for children aged under-two years in LMICs.^20^ We categorised and summarised the interventions extracted; and, when possible, we reported their cost along with their impact on the four domains of motor, cognitive, language and socioemotional skills.

After extracting effectiveness estimates from all studies, we tried to identify cost estimates pertaining to the interventions documented in each study (primary data on costs). For the great majority of studies (13 out of 15 studies), cost estimates (derived from primary data) were not available. Therefore, for those studies, we estimated unit costs of interventions ourselves: to do so, we used an ingredients-based approach to derive a unit cost per child targeted for each intervention reviewed. Since costs were reported from different countries (with very different incomes and wage costs) and across different time periods, we also computed standardised unit cost estimates in 2010 US$ (as studies were conducted from 1991 to 2012) using the World Bank’s consumer price index^21^ and the average gross domestic product per capita for LMICs in the year 2010 (US$3549) as the wage reference point.

## Results

We first briefly summarise the existing cost-effectiveness analysis methods used to evaluate the interventions that promote ECD outcomes. Second, we detail our proposed approach to allow a comparison of the relative cost-effectiveness of these interventions. Third, we apply our approach to compute the effectiveness and cost-effectiveness of the early parenting and psychosocial stimulation interventions reviewed by Aboud and Yousafzai.^19^

### Scoping review: existing ECD cost-effectiveness analysis approaches

Most of the existing cost-effectiveness analysis literature in ECD has focused on benefit-cost analysis, comparing individual increases (for the beneficiaries of these interventions) in later life wages^13 16 22–24^ or societal savings (eg, through reduced incarceration rates) to the short-term cost of intervention for example.^12^ There are three main challenges with this approach: first, estimating long-term benefits is complicated and requires a large number of assumptions, including future growth in wages, discount rates and labour force participation rates that result in a high degree of uncertainty. Second, estimates of long-term benefits to date have been mostly based on a small number of highly effective trials in the 1960s,^12^ 1970s and 1980s,^11^ which may not apply to current interventions. Third, and most importantly, having positive returns to a given intervention does not directly imply that such an intervention should be prioritised if other interventions can achieve similar outcomes at a lower cost.

This challenge has long been recognised in the fields of medical and public health interventions, where clear cost-effectiveness guidelines have been developed over time to directly identify the interventions that yield the highest health gains (eg, deaths or disability-adjusted life years (DALYs) averted) for a given cost.^25 26^ Standard cost-effectiveness analysis and measures such as $ per death or DALY averted cannot however be applied to ECD; interventions that promote ECD are neither designed to improve survival probabilities nor to reduce morbidity (even though such reductions may be achieved by some programmes, either directly or indirectly). Instead, interventions aim at improving children’s cognitive, language and socioemotional development—outcomes that are not captured by DALYs. While improvements in these domains improve children’s early and later life, disability weights to quantify the benefits of these improvements in specific domains of ECD are currently not available.^27 28^

### Drawing from the scoping review: assessing value for money of interventions that promote ECD

With the scoping review of the literature, two important features specific to ECD emerged that need to be considered when assessing the intrinsic impact of interventions. First, ECD is multidimensional. There is a great variety of domains of ECD that have been researched in the literature. Most developmental assessments of children under the age of 3 years focus on four domains: (gross and fine) motor skills; cognitive skills, (expressive and receptive) language skills and socioemotional skills.^29^ While these domains of development can be affected by a single intervention in principle, interventions may have specific focal areas (such as shared book reading on early language or socioemotional development) that are explicitly targeted. Second, improvements in any domain are continuous. Universal interventions designed to promote ECD for all children, such as parenting and stimulation suggest benefits are possible for all children. There is no ‘maximum’ level of development and population-level future improvements in these outcomes should be expected.^30^

With these two critical considerations in mind, we can define the most effective intervention that promotes ECD as one that achieves the largest possible improvements across the four domains of development (denoted *d’s*). To identify the most cost-effective interventions, we can then compare the overall improvements in ECD achieved by an intervention with the required cost. Following traditional cost-effectiveness analysis guidelines,^25 26^ we can compute ‘incremental cost-effectiveness ratios’ (or ICERs) for interventions as: $ICER=\frac{C_{i}-C_{0}}{B_{i}-B_{0}}$, where *C_i_* and *C*_*0*_ are the costs of intervention *i* and of the status quo (ie, the control or standard-of-care group), respectively, and *B_i_* and *B*_*0*_ are the benefits of intervention *i* and of the status quo, respectively.

The incremental nature of the cost calculations (in the ICER computation above) is important because potential intervention programmes can either be implemented on top of existing programmes or replace them, with very different budgetary implications. Benefits can be computed across all four domains in the same way, that is, by comparing the average development of children with the average development in the four domains with status quo. As a result, the full ICER can be expressed as $ICER=\frac{C_{i}-C_{0}}{\Sigma_{d}w_{d}Z_{d,i}-\Sigma_{d}w_{d}Z_{d,0}}$, where *Z*_*d, i*_ is the average standardised developmental outcome in domain *d* with intervention *i*, *Z*_*d,0*_ is the average developmental outcome in the same domain without intervention (status quo) and *w_d_* is the specific weight given to each domain *d*. While all four domains have been associated with improved later life outcomes, policymakers may wish to give higher priority to some domains and rank interventions accordingly. If all domains are given a uniform weight of 1, the denominator of this formula simply becomes the sum of Z-score differences in developmental outcomes with and without the intervention across the four domains. A smaller ICER means a more cost-effective intervention; interventions can thus be rated directly with respect to the ICER computed.

### Review of Aboud and Yousafzai (2015): impact and value for money of interventions that promote ECD

**Figure 1:**
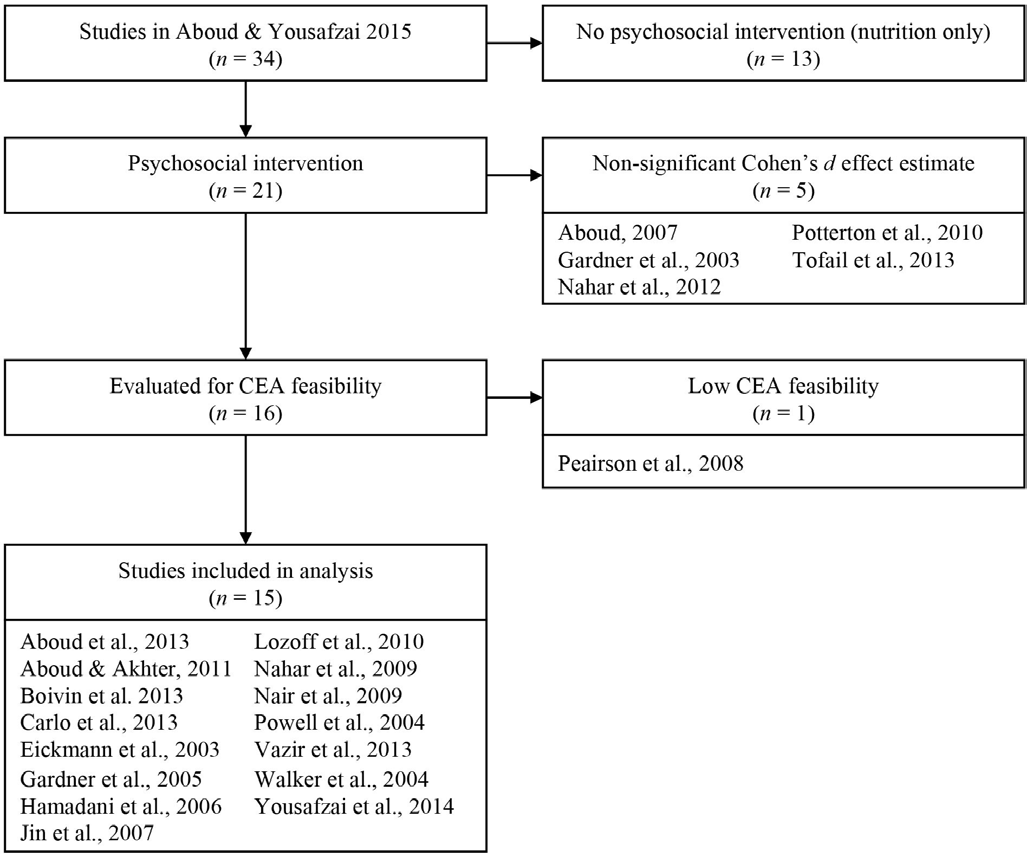
Flow diagram of each study selection process. CEA, cost-effectiveness analysis.

Figure 1 details our study selection process including all the interventions reviewed and retained based off the original review by Aboud and Yousafzai.^19^ Ultimately, 15 studies (out of the 34 studies initially selected)^31 32^ were selected for subsequent analyses. These 15 studies were psychosocial stimulation interventions with direct impact on cognitive and language skills and targeting children between ages 0 and 24 months.

Online supplemental table S1 details the interventions reviewed and provides selected information including a brief description of the intervention arms, the workforce implications, the impact and effectiveness estimates for the two domains of cognitive and language skills (among the four domains identified above) and other benefits and reported cost (if any). The main interventions identified for which we could obtain a developmental impact (estimated in Cohen’s *d* units here) in at least one of the domains included integrated enhanced nutrition and responsive stimulation, child development messages (eg, short films, play materials), home visiting programmes, community-based parenting group sessions and psychosocial stimulation (online supplemental table S1). Only two studies^31 32^ reported cost estimates in addition to a developmental impact.

10.1136/bmjgh-2022-008926.supp1 Supplementary data

We then report on the summarised effectiveness measures and the corresponding estimates of costs using our ingredients-based approach (table 1). We observe a range of effectiveness estimates from −0.41 (95% uncertainty range (UR): −0.82 to 0.00) to 1.80 (95% UR: 1.21 to 2.18), in this case in the cognitive domain from the intervention arms in Lozoff and colleagues (2010) (psychosocial and nutrition intervention with professional educators).^19 33^ As for costs, we computed unit costs varying from $1 per child from Aboud and colleagues (for a psychosocial intervention with home visits delivered by already employed community health workers in Bangladesh)^31^ to $ 2172 per child from Lozoff and colleagues (for an integrated psychosocial and nutrition intervention delivered by professional educators in Chile);^33^ when using standardised costs for LMICs, the unit costs per child were $24 and $3519, respectively.

**Table 1:** Summary of effectiveness (intervention effect that is, Cohen’s *d* effect estimates; 95% uncertainty ranges given in parentheses) and estimated cost (in total and per child targeted) for each intervention that promotes ECD included in this study

Study	Country	Intervention cost		LMIC standardised cost*	Intervention effect	
		Total	Per child	Per child	Domain	Estimate
Aboud and Akhter, 2011^42^	Bangladesh, 2008	$13 151	$69	$99	Cognitive	0.40 (0.10 to 0.69)†
Aboud *et al*, 2013^31^	Bangladesh, 2011		$6	$41	Cognitive	0.67 (0.48 to 0.86)†
Group sessions					Expressive language	0.97 (0.82 to 1.21)†
					Receptive language	0.85 (0.64 to 1.03)†
Aboud *et al*, 2013^31^	Bangladesh, 2011		$1	$24	Cognitive	0.67 (0.48 to 0.86)†
Home visits					Expressive language	0.97 (0.82 to 1.21)†
					Receptive language	0.85 (0.64 to 1.03)†
Boivin *et al*, 2013^43^	Uganda, 2012‡	$39 617	$660	$992	Cognitive	0.03 (−0.33 to 0.40)
					Expressive language	0.39 (0.02 to 0.75)†
					Fine motor	0.11 (−0.25 to 0.47)
					Receptive language	0.44 (0.07 to 0.81)†
Carlo *et al*, 2003^44^	India, Pakistan	$20 487	$347	$1256	Cognitive (non-resuscitated)	0.23 (−0.07 to 0.53)
	Zambia, 2007–2011				Cognitive (resuscitated)	0.37 (0.01 to 0.72)†
Eickmann *et al* 2003^45^	Brazil, 1999	$16 636	$252	$228	Cognitive	0.81 (0.46 to 1.16)†
Gardner *et al*, 2005^46^	Jamaica, 2004‡	$38 333	$782	$1282	Cognitive	0.32 (−0.05 to 0.70)
					Fine motor	0.16 (−0.22 to 0.53)
					Language	0.55 (0.16 to 0.93)†
Hamadani *et al* 2006^34^	Bangladesh, 2000–2002	$16 801	$183	$1357	Cognitive	0.33 (0.04 to 0.61)†
Jin *et al*, 2007^47^	China, 2003	$828	$17	$62	Cognitive	0.48 (0.06 to 0.91)†
					Language	0.51 (0.08 to 0.94)†
Lozoff *et al*, 2010^33^	Chile,1991–1995	$167 228	$2172	$3519	Cognitive (iron-deficient)	1.80 (1.21 to 2.18)†
					Cognitive (non-iron-deficient)	−0.41 (−0.82 to 0.00)
Nahar *et al*, 2009^48^	Bangladesh, 2008‡	$3324	$101	$582	Cognitive	0.84 (0.35 to 1.33)†
Nair *et al*, 2009^49^	India, 2008‡	$1690	$5	$18	Cognitive	0.21 (0.06 to 0.35)†
Powell *et al*, 2004^50^	Jamaica, 2003‡	$50 753	$781	$1290	Cognitive	0.87 (0.87 to 1.23)†
					Fine motor	0.71 (0.35 to 1.07)†
					Language	0.77 (0.41 to 1.13)†
Vazir *et al*, 2013^51^	India, 2012‡	$22 534	$147	$418	Cognitive	0.36 (0.14 to 0.57)†
Walker *et al*, 2004^52^	Jamaica, 2003‡	$28 218	$448	$735	Cognitive	0.42 (0.07 to 0.77)†
					Language	0.00 (−0.34 to 0.34)
Yousafzai *et al*, 2014§^32^	Pakistan, 2009–2012	$48 816	$134	$134	Cognitive	0.60 (0.45 to 0.76)†
					Language	0.70 (0.45 to 0.75)†

*Hypothetical cost of interventions if conducted in a typical LMIC in 2010 (uses the average gross domestic product per capita for LMICs in the year 2010 (US$3549) as the wage reference point). Workforce costs were modified; however, additional input costs (ie, books, toys etc.) were not, as the estimates used were already standardised across interventions.

†Statistically significant.

‡Study year not explicitly stated; using year prior to article publication.

§Cost inputs extracted from the linked study by Gowani *et al*.^53^

ECD, early childhood development; LMIC, low-income and middle-income country; RCT, randomised controlled trial.

### Applying the economic evaluation framework

Combining cost and effectiveness measures, we were able to assess value for money, that is, to compute ICERs for 12 interventions (table 2). These value-for-money (or cost-effectiveness) estimates enable ranking and possible prioritisation of interventions: in other words, having positive returns to a given intervention does not directly imply that such an intervention should be prioritised if other interventions can achieve similar outcomes at a lower cost. These cost-effectiveness estimates can help prioritise those interventions with greater returns on ECD outcomes for similar budget expenditure impact. When using an averaged effect estimate (mean of the effect estimates across the domains for which an effect size was reported), the two most cost-effective interventions were the psychosocial interventions by Aboud and colleagues (home visits) and Aboud and colleagues (group sessions),^31^ with ICERs of $29 (95% UR: $23–$37) and $49 ($40–$63) per SD increase in domain-specific ECD, respectively (using LMIC-standardised costs), while the least cost-effective interventions would be the psychosocial intervention by Hamadani and colleagues^34^ and the psychosocial and nutrition intervention with professional educators by Lozoff and colleagues,^33^ with ICERs of $4112 ($2225–33 925) and $5063 ($3228–18 046) per SD increase in domain-specific ECD, respectively. When using the combined effect estimate (sum of the effect estimates across the domains), the most cost-effective interventions remained the same, with ICERs between $10 ($8–$12) and $16 ($13–$21) per SD increase in domain-specific ECD. The least cost-effective interventions were also the same, with ICERs between $2532 ($1614–$9023) and $5063 ($3228–$18 046) per SD increase in domain-specific ECD, respectively.

**Table 2:** Summary of estimated cost-effectiveness (ICER, 95% uncertainty ranges given in parentheses) for each intervention that promotes ECD included in this study

Study	Effect estimate	Standardised cost-effectiveness			Intervention cost-effectiveness (local cost)		
		Cost per child	Rank	Cost-effectiveness (ICER)	Cost per child	Rank	Cost-effectiveness (ICER)
Averaged effect estimate							
Aboud *et al*, 2013 (Home visits)^31^	0.83 (0.65 to 1.03)	$24	1	$29 ($23 to $37)	$1	1	$1 ($1 to $2)
Aboud *et al*, 2013 (Group sessions)^31^	0.83 (0.65 to 1.03)	$41	2	$49 ($40 to $63)	$6	2	$7 ($6 to $9)
Nair *et al*, 2009^49^	0.21 (0.06 to 0.35)	$18	3	$86 ($51 to $300)	$5	3	$24 ($14 to $83)
Jin *et al*, 2007^47^	0.50 (0.07 to 0.92)	$62	4	$125 ($67 to $886)	$17	4	$34 ($18 to $243)
Yousafzai *et al*, 2014^32^	0.65 (0.45 to 0.76)	$134	5	$206 ($177 to $298)	$134	7	$206 ($177 to $298)
Aboud and Akhter, 2011^42^	0.40 (0.10 to 0.69)	$99	6	$248 ($143 to $990)	$69	6	$172 ($100 to $690)
Eickmann *et al*, 2003^45^	0.81 (0.46 to 1.16)	$228	7	$281 ($197 to $496)	$252	8	$311 ($217 to $548)
Nahar *et al*, 2009^48^	0.84 (0.35 to 1.33)	$582	8	$693 ($438 to $1663)	$101	5	$120 ($76 to $289)
Vazir *et al,* 2013^51^	0.36 (0.14 to 0.57)	$418	9	$1161 ($733 to $2986)	$147	9	$408 ($258 to $1050)
Powell *et al,* 2004^50^	0.78 (0.54 to 1.14)	$1290	10	$1647 ($1128 to $2374)	$781	11	$997 ($683 to $1437)
Hamadani *et al,* 2006^34^	0.33 (0.04 to 0.61)	$1357	11	$4112 ($2225 to $33,925)	$183	10	$555 ($300 to $4575)
Lozoff *et al,* 2010^33^	0.70 (0.20 to 1.09)	$3519	12	$5063 ($3228 to $18,046)	$2172	12	$3125 ($1993 to $11 138)
Summed effect estimate							
Aboud *et al*, 2013 (Home visits)^31^	2.49 (1.94 to 3.10)	$24	1	$10 ($8 to $12)	$1	1	$0 ($0 to $1)
Aboud *et al,* 2013 (Group sessions)^31^	2.49 (1.94 to 3.10)	$41	2	$16 ($13 to $21)	$6	2	$2 ($2 to $3)
Jin *et al,* 2007^47^	0.99 (0.14 to 1.85)	$62	3	$63 ($34 to $443)	$17	3	$17 ($9 to $121)
Nair *et al,* 2009^49^	0.21 (0.06 to 0.35)	$18	4	$86 ($51 to $300)	$5	4	$24 ($14 to $83)
Yousafzai *et al,* 2014^32^	1.30 (0.90 to 1.51)	$134	5	$103 ($89 to $149)	$134	5	$103 ($89 to $149)
Aboud and Akhter, 2011^42^	0.40 (0.10 to 0.69)	$99	6	$248 ($143 to $990)	$69	7	$172 ($100 to $690)
Eickmann *et al,* 2003^45^	0.81 (0.46 to 1.16)	$228	7	$281 ($197 to $496)	$252	8	$311 ($217 to $548)
Powell *et al,* 2004^50^	2.35 (1.63 to 3.43)	$1290	8	$549 ($376 to $791)	$781	9	$332 ($228 to $479)
Nahar *et al,* 2009^48^	0.84 (0.35 to 1.33)	$582	9	$693 ($438 to $1663)	$101	6	$120 ($76 to $289)
Vazir *et al,* 2013^51^	0.36 (0.14 to 0.57)	$418	10	$1161 ($733 to $2986)	$147	10	$408 ($258 to $1050)
Lozoff *et al,* 2010^33^	1.39 (0.39 to 2.18)	$3519	11	$2532 ($1614 to $9023)	$2172	12	$1563 ($996 to $5,569)
Hamadani *et al*, 2006^34^	0.33 (0.04 to 0.61)	$1357	12	$4112 ($2225 to $33,925)	$183	11	$555 ($300 to $4,575)

Averaged effect estimate=mean of the effect estimates across the domains of cognitive and language skills for which an effect size was reported in the study.

Summed effect estimate=sum of the effect estimates across the two domains of cognitive and language skills, for which an effect size was reported in the study.

Standardised cost-effectiveness=uses the average gross domestic product per capita for LMICs in the year 2010 (US$3549) as the wage reference point.

Note that the status quo and time horizon in the calculation of ICERs corresponds to the status quo and time horizon retained in each of the studies for which an ICER was computed.

ECD, early childhood development; ICER, incremental cost-effectiveness ratio; LMICs, low-income and middle-income countries.

## Discussion

With increased attention and global funding towards ECD,^35 36^ an analytical framework for ranking interventions that promote ECD across settings will be needed. In this paper, we have introduced a basic value-for-money approach designed to make interventions directly comparable across settings, and we show that the computed ICERs would vary widely across settings.

As it is often the case for health interventions, cost-effectiveness rankings are dominated by costing aspects. Rather remarkably, we found the cost ratio of the most expensive relative to the cheapest intervention exceeded 100:1, while impacts on ECD across a rather diverse set of interventions were relatively similar. Unsurprisingly, the most cost-effective interventions were those with the lowest cost of implementation; the most cost-effective intervention analysed was a psychosocial stimulation intervention implemented with close to zero additional staff resources in Bangladesh.^31^ (In this study, group sessions were delivered by already employed (uncompensated) village peer educators, while home visits were delivered by community health workers who would normally visit households to talk about health. In this respect, the use of volunteers in this study might face challenges at scale such as staff retention.) Lower ICERs associated with higher costs of implementation do not necessarily mean that the returns to these programmes will not be high (see the case for instance of the psychosocial and nutrition intervention in Chile^33^). In addition, many analyses have shown that both private and social returns to ECD programmes in high-income countries (where implementation costs are higher) are rather substantial.

This is to our knowledge the first attempt to rank different interventions across countries, and evidently, it comes with a number of important limitations. First, we only could source a few studies of interventions that would comprehensively report on intervention impact in a consistent manner across our choice of ECD outcomes (figure 1; online supplemental table S1). As a result, we had little coverage of all possible interventions and settings in which they could be implemented. Second, we used a standardised measure for intervention effect (using Cohen’s *d*) across studies, while there is some variety in the measures used to report ECD outcomes per intervention (eg, Bayley scales of infant development, McCarthy score). While the Bayley scales for infant and toddler development^37^ are considered a gold standard by many researchers, the tool was developed originally for high-income settings and requires formative work to ensure appropriate adaptation, reliability and validity for application in diverse cultural settings (often the tool is not adapted and the psychometric properties are not commonly reported). A large number of tools have been used that measure developmental outcomes in different ways (eg, direct child assessment, caregiver report) that may not be directly comparable. Efforts to develop a measure of development for children less than 3 years of age that can be relatively less of a burden on time and open access are underway, including the global scale for early development tool that may make these comparisons easier in the future.^38 39^ Third, aggregation of ECD outcomes (eg, cognitive skills added to language skills) within one study could well be limited by potentially high correlations across ECD outcomes in the study, and beyond mere additions, there may be reinforcing multiplicative effects between ECD outcomes. Fourth, the likely long-term effects of interventions were not included in our modelling: for example, interventions that promote ECD may improve human capital outcomes including greater educational attainment and potentially higher wages into future adulthood (with very significant returns). Also, we did not discount ECD benefits and costs in our computed ICERs, even though this could be further done using standard economic evaluation guidelines.^25 26^ Likewise, we focused on ECD-specific interventions, that is, interventions that primarily target ECD outcomes. As such, we did not include in our review ‘ECD-sensitive’ interventions, that is, interventions that promote ECD indirectly, say, for example, infant and young child feeding promotion, or child multiple micronutrient supplementation (which is designed to reduce anaemia and micronutrient deficiencies), for which a recent meta-analysis of the effects on ECD was published by Prado and colleagues.^40^ Fifth, given the extreme scarcity of data, our quantitative findings should be interpreted with caution before any recommendation can be drawn. In this respect, important considerations including the distribution in ECD outcomes at the population level or across socioeconomic status (eg, wealth and education levels) as well as more generally any distributional or equity aspects of the ECD interventions were not taken into account; neither were possible mediating effects of the environment (eg, classroom quality) for ECD.^41^ Last, we envision future work would strengthen consistent and comparable measurement on data collection on the effectiveness side, tied with rigorous economic data on the implementation cost side so that value-for-money arguments for priority setting can be developed. We intend that the work presented will be a first step towards a unified analytical framework that will stimulate conversations on assessment of the relative importance and cost-effectiveness of ECD interventions at the national and global levels. In this respect, fully including ECD outcomes into priority setting is now an urgent necessity.

## Data Availability

All data relevant to the study are included in the article or uploaded as supplementary information.
