# Supplementary material for: Priority setting in early childhood development: an analytical framework for economic evaluation of interventions
Source: BMJ Glob Health. 2022 Jun 20;7(6):e008926. doi: 10.1136/bmjgh-2022-008926 (PMC9214350; doi:10.1136/bmjgh-2022-008926)
Supplement: Supplementary data [file bmjgh-2022-008926supp001.pdf]

Supplementary webappendix

Table S1. Study information: details on the selected interventions that promote early childhood development (ECD), including: intervention arms; workforce requirements; impact and effectiveness estimates, for the two domains of cognitive skills and language skills; and reported cost (if any).

Study context	Intervention arms	Workforce	Additional inputs	Effect estimates*	Reported cost
Aboud & Akhter 2011					
Bangladesh<br>2008<br><br>Cluster randomized field trial.<br><br>Peer educator demonstrated responsive stimulation and feeding activities with 1 child and then coached mothers as they practiced with their own child. Mothers were asked to bring a play bag and to gradually fill it with home materials for play.	*Combined and costed*<br>1. Psychosocial<br>– 92 children<br>– Responsive feeding and stimulation (RSF)<br>– 6 group sessions with trainees<br>2. Psychosocial + Nutrition<br>– 100 children<br>– Responsive feeding and stimulation with 6-month supply fortified food powder (RSF+)<br>– 6 group sessions with trainees<br>*Not costed*<br>3. Control	1. Trainees<br>– Peer educators<br>– 4 days training	1. Flip charts<br>– Both arms<br>2. 6-month supply fortified food powder<br>– Psychosocial + Nutrition	1. Cognitive<br>– INT 32.7 ± 21.3<br>– CTRL 24.9 ± 18.2<br>– Cohen's *d* = 0.40 (0.10, 0.69)	No costs reported

Aboud et al. 2013					
Bangladesh<br>2011<br><br>Cluster-stratified field trial.<br>In one intervention arm, peer educators used an illustrative card showing key practices; cards were given to caregivers. In the other, family welfare assistants delivered messages during counselling sessions to mothers at home and community clinics.	***Costed (combined)***<br>1. Psychosocial A (group sessions)<br>– 113 children<br>– 14 group sessions with volunteers<br>2. Psychosocial B (home visits)<br>– 113 children<br>– 2 home visits with paraprofessionals<br>***Not costed***<br>3. Control	1. Volunteers<br>– Unpaid peer educators<br>– 7 days training<br>– 18 families per volunteer<br>2. Paraprofessionals<br>– Family welfare assistants<br>– 3 days training<br>– $100 (monthly salary + per diem)<br>– 600 families per family welfare assistant	1. Illustrative cards<br>– Psychosocial A	1. Cognitive<br>– INT 60.7 ± 5.7<br>– CTRL 56.1 ± 7.9<br>– Cohen's *d* = 0.67 (0.48, 0.86)<br>2. Receptive language<br>– INT 27.1 ± 3.7<br>– CTRL 22.7 ± 6.4<br>– Cohen's *d* = 0.85 (0.64, 1.03)<br>3. Expressive language<br>– INT 29.6 ± 4.8<br>– CTRL 3.8 ± 6.5<br>– Cohen's *d* = 0.97 (0.82, 1.21)	1. Psychosocial A (group sessions)<br>– $6.17 per child<br>2. Psychosocial B (home visits)<br>– $1.34 per child
Boivin et al. 2013					
Uganda<br>Unknown year<br><br>RCT.<br><br>25 home visits fortnightly for 12 months with instruction, demonstration, and practice in how to interact with child.	***Costed***<br>1. Psychosocial<br>– 60 children<br>– 26 home visits and clinic sessions with trainees<br>***Not costed***<br>2. Control	1. Trainees	1. Video equipment	1. Receptive language<br>– INT 35 ± 9.36[**]<br>– CTRL 33 ± 9.64[**]<br>– Cohen's *d* = 0.44 (0.07, 0.81)<br>2. Expressive language<br>– INT 35 ± 10.71[**]<br>– CTRL 32 ± 10.39[**]<br>– Cohen's *d* = 0.39 (0.02, 0.75)	No costs reported

Carlo et al. 2013					
India, Pakistan, Zambia<br>2007-2011<br><br>Parallel group RCT.<br><br>Peer educators introduced and modeled playful interactive learning activities depicted on illustrative cards; cards were given to caregivers.	*Costed*<br>1. Psychosocial (resuscitated)<br>– 59 children<br>– 68 home visits with trainees<br>*Not costed*<br>2. Psychosocial (non-resuscitated)<br>3. Control A (resuscitated)<br>4. Control B (non-resuscitated)	1. Trainees<br>– Peer educators<br>– 10 days training	1. Illustrative cards	1. Cognitive<br>– INT 102.6 ± 9.8<br>– CTRL 98.0 ± 14.6<br>– Cohen's *d* = 0.37 (0.01, 0.72)	No costs reported
Eickmann et al. 2003					
Brazil<br>1999<br><br>Non-randomized cluster trial.<br><br>Initial home visit Professional explained the importance of child play and interaction and invited mother to participate in program during first home visit. Group sessions held to introduce and practice concepts; home visits reinforced what had been taught.	*Costed*<br>1. Psychosocial<br>– 66 children (at 18-month evaluation)<br>– 1 home visit with professionals<br>– 3 group sessions with professionals (8 mothers per session)<br>– 10 home visits with trainees<br>*Not costed*<br>2. Control	1. Professionals<br>– 2 occupational therapists<br>2. Trainees<br>– 5 peer educators	1. Toys, homemade<br>– 1 per child per home visit	1. Cognitive<br>– INT 105.9 ± 8.8<br>– CTRL 95.6 ± 13.7<br>– Cohen's *d* = 0.81 (0.46, 1.16)	No costs reported

Gardner et al. 2005					
Jamaica<br>Unknown year<br><br>Cluster RCT.<br><br>Stimulation program comprised weekly home visits during which play was demonstrated and maternal-child interactions were encouraged.	***Costed (combined)***<br>1. Psychosocial<br>– 23 children<br>– 24 home visits with paraprofessionals<br>2. Psychosocial + Nutrition<br>– 26 children<br>– 24 home visits with paraprofessionals<br>***Not costed***<br>3. Nutrition<br>4. Control	1. Paraprofessionals<br>– Community health workers	1. Toys, homemade<br>– Kept for 1 week and returned<br>2. 6-month supply zinc sulfate<br>– Psychosocial + Nutrition	1. Language<br>– INT 100.4 ± 13.7**<br>– CTRL 93.3 ± 12.5**<br>– Cohen's *d* = 0.55 (0.16, 0.93)	No costs reported
Hamadani et al. 2006					
Bangladesh<br>2000-2002<br><br>Cluster RCT.<br><br>Group sessions included topics on child development and the importance of play. During home visits, play leaders demonstrated activities to the mothers.	***Costed***<br>1. Psychosocial (undernourished)<br>– 92 children<br>– 39 group sessions with trainees<br>– 86 home visits with trainees<br>***Not costed***<br>2. Control (undernourished)<br>3. Control (better nourished)	1. Trainees<br>– Play leaders<br>– 2 weeks training	1. Picture books<br>2. Toys, homemade<br>– Kept for 1 week and returned	1. Cognitive<br>– INT 87.6 ± 13.3<br>– CTRL 82.7 ± 17.2<br>– Cohen's *d* = 0.33 (0.04, 0.61)	No costs reported

**Jin et al. 2007**					
China<br>2003<br><br>RCT.<br><br>Mothers received counseling using the Care for Development (CFD) Mother's Card and were provided with a copy.	***Costed***<br>1. Psychosocial<br>– 50 children<br>– 2 sessions with professionals<br>***Not costed***<br>2. Control	1. Professionals<br>– Health professionals trained by WHO	1. Illustrative card<br>– 1 per child	1. Cognitive<br>– INT 93.69 ± 9.42<br>– CTRL 88.69 ± 11.28<br>– Cohen's *d* = 0.48 (0.06, 0.91)<br>2. Language<br>– INT 96.62 ± 12.42<br>– CTRL 90.33 ± 12.13<br>– Cohen's *d* = 0.51 (0.08, 0.94)	No costs reported
**Lozoff et al., 2010**					
Chile<br>1991-1995<br><br>Parallel group RCT.<br><br>Intervention visits were conducted by professional educators devoted to encouraging the development of individual abilities.	***Costed***<br>1. Psychosocial + Nutrition (iron deficient)<br>– 77 children (combined iron deficient 6- and 12-month arms)<br>– 52 home visits with professionals<br>***Not costed***<br>2. Psychosocial + Nutrition (non-iron deficient, 6- and 12-month arms)<br>3. Control (iron deficient)<br>4. Control (non-iron deficient)	1. Professionals<br>– Professional educators<br>– Visited 6 families per week	1. 6-month supply oral iron	1. Cognitive (iron deficient)<br>– INT 134.57 ± 7.0**<br>– CTRL 121.9 ± 7.1**<br>– Cohen's *d* = 1.8 (1.21, 2.18)	No costs reported

Nahar et al. 2009					
Bangladesh<br>Unknown year<br><br>Time-lagged non-randomized trial.<br><br>Group and individual play sessions with child at clinic; home visits for 6 months with discussions on play and talk.	***Costed***<br>1. Psychosocial<br>– 33 children<br>– 7 facility sessions with paraprofessionals<br>– 11 home visits with paraprofessionals<br>***Not costed***<br>2. Control	1. Paraprofessionals<br>– Health workers<br>– 2 weeks training	1. Toys, homemade<br>– Kept for 1 week and returned	1. Cognitive<br>– INT 103.1 ± 12.1<br>– CTRL 94.3 ± 8.8<br>– Cohen's *d* = 0.84 (0.35, 1.33)	No costs reported
Nair et al. 2009					
India<br>Unknown year<br><br>Child Development Centre model whereby an occupational therapist trained mothers to provide stimulation and encouraged them to continue practice at home.	***Costed***<br>1. Psychosocial<br>– 324 children<br>– 1 facility visit with professional<br>***Not costed***<br>2. Control	1. Professionals<br>– Occupational therapists	N/A	1. Cognitive (at 2 years)<br>– INT 83.1 ± 13.9<br>– CTRL 80.3 ± 13.4<br>– Cohen's *d* = 0.21 (0.06, 0.35)	No costs reported

**Powell et al. 2004**					
Jamaica<br>Unknown year<br><br>Cluster RCT.<br><br>Weekly home visits by community health aides for one year in addition to usual duties. Parenting issues were discussed with the mothers and play activities were demonstrated with the children using homemade materials.	***Costed***<br>1. Psychosocial<br>– 65 children<br>– 52 home visits with paraprofessionals<br>***Not costed***<br>2. Control	1. Paraprofessionals<br>– Community health aides<br>– 2 weeks training<br>– 3-5 children per day	1. Toys, homemade<br>– Kept for 1 week then returned<br>2. Picture books	1. Cognitive<br>– INT 94.5 ± 15.3<br>– CTRL 83.0 ± 10.9<br>– Cohen's *d* = 0.87 (0.87, 1.23)<br>2. Language<br>– INT 100.7 ± 15.4<br>– CTRL 89.4 ± 13.9<br>– Cohen's *d* = 0.77 (0.41, 1.13)<br>3. Fine motor<br>– INT 97.6 ± 10.7<br>– CTRL 90.6 ± 8.9<br>– Cohen's *d* = 0.71 (0.35, 1.07)	No costs reported
**Vazir et al. 2013**					
India<br>Unknown year<br><br>Cluster RCT.<br><br>Received education on complementary feeding, messages and skills on responsive feeding, and developmental stimulation messages using five simple toys.	***Costed***<br>1. Psychosocial + nutrition<br>– 153 children<br>– Responsive complementary feeding and play group (RCF&PG)<br>– 30 home visits with trainees<br>***Not costed***<br>2. Nutrition<br>– Responsive complementary feeding (CFG)<br>3. Control	1. Trainees<br>– 60 village women	1. Flip charts<br>2. Toys<br>– 5 per child	1. Cognitive<br>– INT 107.4 ± 8.1<br>– CTRL 104.4 ± 8.6<br>– Cohen's *d* = 0.36 (0.14, 0.57)	No costs reported

**Walker et al. 2004**					
Jamaica<br>Unknown year<br><br>RCT.<br><br>Weekly home visits demonstrating play and child interactions for 25 weeks (5-month space between first and second phases of interventions).	***Costed***<br>1. Psychosocial (low birth weight)<br>– 63 children<br>– 25 home visits with paraprofessionals<br>***Not costed***<br>2. Control (low birth weight)<br>3. Control (normal birth weight)	1. Paraprofessionals<br>– 3 community health workers<br>– 2 weeks training	1. Toys, homemade<br>– Kept for 1 week and returned	1. Cognitive<br>– INT 87.0 ± 9.3<br>– CTRL 83.1 ± 9.2<br>– Cohen's *d* = 0.42 (0.07, 0.77)	No costs reported
**Yousafzai et al. 2014**					
Pakistan<br>2009-2012<br><br>Cluster RCT, 2x2 factorial design.<br><br>Stimulation integrated in routine home and group meetings delivered by government Lady Health Workers.	***Costed***<br>1. Psychosocial<br>– 364 children<br>– Monthly home visits and monthly community sessions<br>***Not costed***<br>2. Psychosocial + Nutrition<br>3. Nutrition<br>4. Control	1. Paraprofessionals<br>– Lady Health Workers; only "incentive" payments beyond standard government salary<br>– Visit 5-7 households per day<br>– 3 days training + 1 refresher every 6 months	1. Picture book<br>– 1 per child	1. Cognitive<br>– INT 83.2 ± 19.3**<br>– CTRL 71.9 ± 18.0**<br>– Cohen's *d* = 0.60 (0.45, 0.76)<br>2. Language<br>– INT 86.1 ± 17.1**<br>– CTRL 75.7 ± 17.5**<br>– Cohen's *d* = 0.70 (0.45, 0.75)	No costs reported (besides Lady Health Worker cost, indicated under workforce)

INT = intervention; CTRL = control; RCT = randomized controlled trial.
*All Cohen's *d* effect estimates extracted from Aboud & Yousafzai (2015).
**INT and CTRL estimates extracted from Aboud & Yousafzai (2015).
